# Improved catheter tracking during cardiovascular magnetic resonance-guided cardiac catheterization using overlay visualization

**DOI:** 10.1186/s12968-022-00863-3

**Published:** 2022-06-02

**Authors:** Joshua S. Greer, Mohamed Abdelghafar Hussein, Ravi Vamsee, Yousef Arar, Sascha Krueger, Steffen Weiss, Jeanne Dillenbeck, Gerald Greil, Surendranath R. Veeram Reddy, Tarique Hussain

**Affiliations:** 1 Department of Pediatrics, University of Texas Southwestern Medical Center, 5323 Harry Hines Blvd, Dallas, TX 75390 USA; 2 Department of Radiology, University of Texas Southwestern Medical Center, 5323 Harry Hines Blvd, Dallas, TX 75390 USA; 3 Advanced Imaging Research Center, University of Texas Southwestern Medical Center, 5323 Harry Hines Blvd, Dallas, TX 75390 USA; 4 Pediatric Department, Kafrelsheikh University, Kafr el-Sheikh, Egypt; 5 Philips Research Laboratories, Philips GmbH Innovative Technologies, Hamburg, Germany; 6 Children’s Medical Center Dallas, 1935 Medical District Drive, Dallas, TX 75235 USA

**Keywords:** Invasive CMR, Congenital heart disease, Pediatrics, Cardiac catheterization

## Abstract

**Introduction:**

Cardiovascular magnetic resonance (CMR)-guided cardiac catheterization is becoming more widespread due to the ability to acquire both functional CMR measurements and diagnostic catheterization data without exposing patients to ionizing radiation. However, the real-time imaging sequences used for catheter guidance during these procedures are limited in resolution and the anatomical detail they can provide. In this study, we propose a passive catheter tracking approach which simultaneously improves catheter tracking and visualization of the anatomy.

**Methods:**

60 patients with congenital heart disease underwent CMR-guided cardiac catheterization on a 1.5T CMR scanner (Ingenia, Philips Healthcare, Best the Netherlands) using the Philips iSuite system. The proposed T1-overlay technique uses a commercially available heavily T1-weighted sequence to image the catheter, and overlays it on a high-resolution 3D dataset within iSuite in real-time. Suppressed tissue in the real-time images enables the use of a thick imaging slab to assist in tracking of the catheter. Improvement in catheter visualization time was compared between T1-overlay and the conventional invasive CMR (iCMR) balanced steady state free precession (bSSFP) sequence. This technique also enabled selective angiography visualization for real-time evaluation of blood flow dynamics (such as pulmonary transit time), similar to direct contrast injection under standard fluoroscopy. Estimates of pulmonary transit time using iCMR were validated using x-ray fluoroscopy in 16 patients.

**Results:**

The T1-overlay approach significantly increased the time that the catheter tip was kept in view by the technologist compared to the bSSFP sequence conventionally used for iCMR. The resulting images received higher ratings for blood/balloon contrast, anatomy visualization, and overall suitability for iCMR guidance by three cardiologists. iCMR selective angiography using T1-overlay also provided accurate estimates of pulmonary transit time that agreed well with x-ray fluoroscopy.

**Conclusion:**

We demonstrate a new passive catheter tracking technique using the iSuite platform that improves visualization of the catheter and cardiac anatomy. These improvements significantly increase the time that the catheter tip is seen throughout the procedure. We also demonstrate the feasibility of iCMR selective angiography for the measurement of pulmonary transit time.

**Supplementary Information:**

The online version contains supplementary material available at 10.1186/s12968-022-00863-3.

## Background

Cardiac catheterization using x-ray fluoroscopy is routinely performed in the clinical management of patients with congenital heart disease. These pediatric patients often require multiple catheterization procedures and receive high cumulative doses of radiation over their lifetime, which significantly increases their risk of developing cancer [1, 2]. Due to this increased exposure to radiation, there has been growing interest in the use of invasive cardiovascular magnetic resonance (iCMR) as a radiation-free alternative for catheter guidance [3–11].

Real-time imaging for catheter guidance during iCMR procedures requires a high frame rate and the ability to visualize the catheter and cardiac structures simultaneously. This is commonly performed using a balanced steady-state free precession (bSSFP) acquisition, which provides bright signal from a gadolinium filled balloon-tip catheter and the blood pool. A partial saturation (pSAT) pre-pulse is often applied to increase the contrast between the blood pool and balloon [7, 8]. However, due to the high frame rate required for catheter guidance, such passive tracking approaches are limited in the spatial resolution and anatomical detail that they can provide. These low-resolution acquisitions also have reduced signal-to-noise ratio (SNR) due to the partial saturation pulse, which can impede catheter navigation in some cases. Additionally, the slice thickness of these real-time imaging sequences is usually limited to around 8–10 mm to preserve anatomical detail, which often results in the loss of balloon visualization as the catheter is advanced through the vasculature, which can prolong the procedure. Therefore, improved simultaneous visualization of both the catheter tip and the patient’s anatomy would be beneficial for iCMR-guided catheterization.

The purpose of this study was to demonstrate visualization of the catheter tip by overlaying isolated T1-weighted gadolinium balloon signal on a static, high-resolution 3D whole-heart dataset in real-time for improved catheter tracking (T1-overlay). Additionally, because the T1-overlay approach enables excellent visualization of the gadolinium contrast agent and its location within the vasculature, it also enabled selective CMR angiography to be performed with anatomical context to the dynamic contrast-enhanced images. Selective angiography is routine in the fluoroscopy lab for the real-time evaluation of blood flow dynamics for the identification of shunts and pulmonary arteriovenous malformations (AVMs), and the development of a suitable alternative in the iCMR suite could minimize unnecessary transfer to the catheterization lab and accelerate the adoption of iCMR. In this study, selective angiography was demonstrated using the T1-overlay technique for the measurement of pulmonary transit time.

## Methods

Sixty patients with congenital heart disease (7 ± 5 years, 53 single ventricle) who underwent iCMR-guided cardiac catheterization under general anesthesia were scanned using the T1-overlay approach on a 1.5T CMR scanner (Ingenia, Philips Healthcare, Best, the Netherlands) at Children’s Medical Center in Dallas, Texas, USA. All patients were consecutively enrolled, had clinical indications for cardiac catheterization and CMR evaluation, and were scanned with IRB approval (STU 032017-061) and informed consent.

In each patient, a 3D bSSFP whole-heart dataset was acquired at the beginning of the procedure to be used as a roadmap for real-time navigation. Following the routine clinical CMR protocol, such as standard 2D cines and flows, the catheterization procedure is performed to acquire pressure and oxygen saturation measurements. The Philips iSuite platform (Philips Research, Hamburg, Germany) [12] was used for real-time catheter guidance, and provided simultaneous visualization of up to three imaging planes with interactive control of the slice geometry throughout the exam. A 6-French Arrow, balloon wedge-pressure catheter (Teleflex Medical, Wayne, Pennsylvania, USA) was used for all procedures. The balloon-tip of the catheter was filled with dilute gadolinium (1-part gadolinium to 99-parts saline) for visualization while obtaining hemodynamic measurements. Catheterization was performed before the administration of any contrast agent to maintain high contrast between the blood pool and the gadolinium -filled balloon. An CMR-conditional guidewire was used to assist in accessing some cardiac structures (angled-tip Emeryglide MRWire, Nano4Imaging, Aachen, Germany). At the tip of the guidewire, three nanoparticle-coated segments produce distinct susceptibility artifacts for passive visualization [7].

The 3D bSSFP roadmap dataset was acquired with 1.8 mm isotropic resolution, TE/TR = 1.8/3.6 ms, SENSE factor = 1.5 in both phase and slice directions, flip angle = 60°, with fat suppression, a T2-preparation, end-systolic electrocardiogram (ECG) triggering, and a diaphragmatic respiratory navigator with a 0.6 tracking factor and 5-mm window to reduce respiratory motion artifacts. Since these whole-heart datasets are frequently acquired for the clinical assessment of patients with congenital heart disease [13], acquiring this 3D roadmap adds no additional time to the iCMR procedure. 3D turbo spin echo (TSE) black-blood images are also acquired in many of these patients to improve the success rate in diagnosing anatomical abnormalities [14], particularly for pulmonary vein imaging [15] or near metallic implants. These images were used as an alternative 3D roadmap in some patients, when they provided superior anatomical visualization.

Real-time imaging was performed using both the established bSSFP pSAT sequence [11] and T1-overlay sequence in the first 30 patients, providing a direct comparison of the two approaches. To evaluate the ability of the technologist to maintain visualization of the catheter with each technique, the imaging sequence used at the beginning each procedure was randomized across patients and alternated for different portions of the procedure (for example: bSSFP for right heart catheterization and T1-overlay for pulmonary artery catheterization). This was done to minimize the effects of catheter heating or anatomical variations influencing the assessment of catheter visualization time.

### bSSFP pSAT

**Fig. 1:**
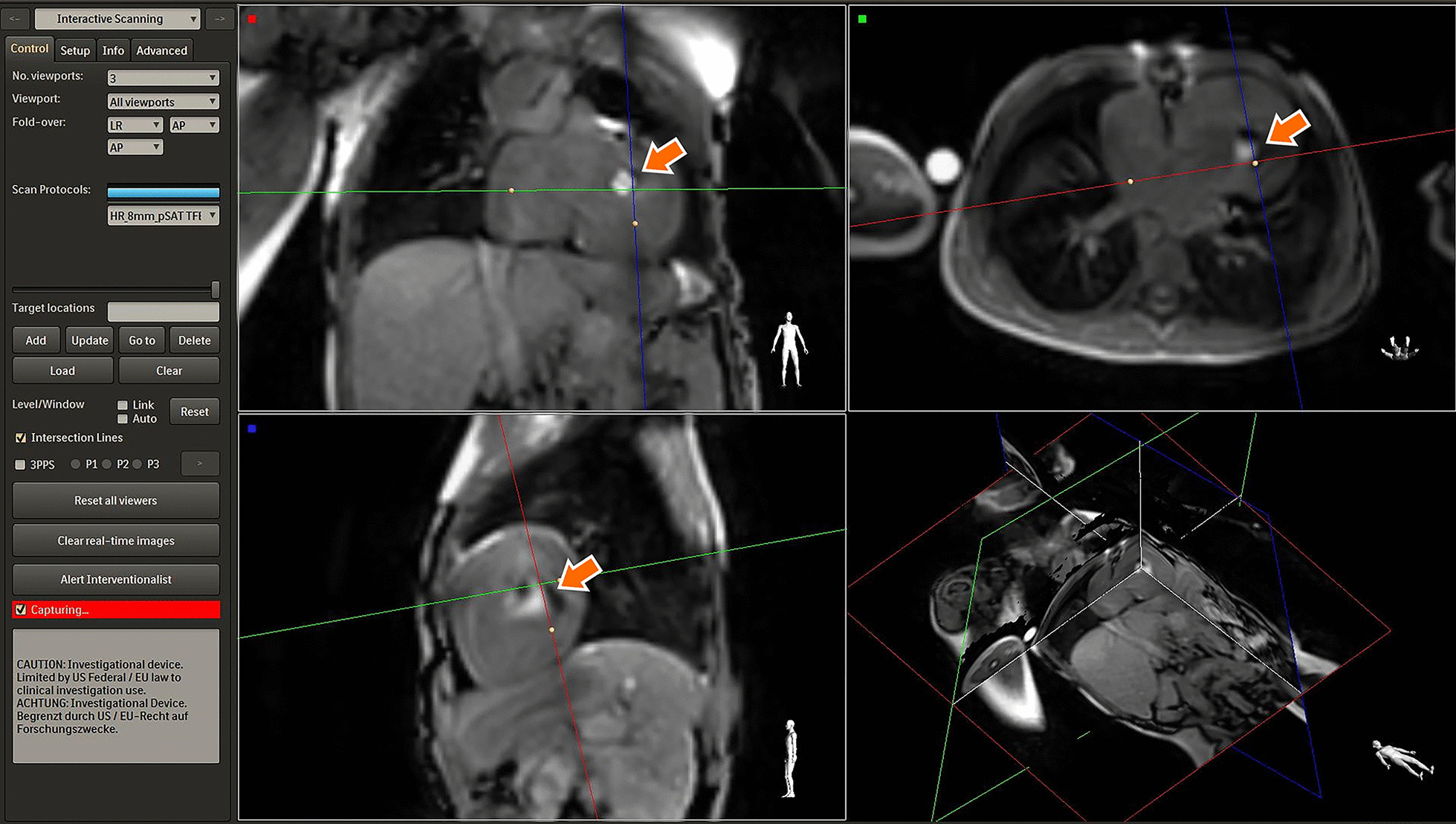
The iSuite (Philips Healthcare, Best, the Netherlands) platform with the previously proposed balanced steady state free precession (bSSFP) partial saturation (pSAT) acquisition in 3 imaging planes. The catheter can be seen in the left ventricle of this patient (arrows)

The pSAT approach was implemented as previously described [7, 11], with a 2D bSSFP acquisition with slice thickness = 8 mm, TE/TR = 1.4/2.8 ms, a 40° nonselective partial saturation pre-pulse to increase the contrast between the gadolinium balloon and surrounding blood pool. The field of view was 350 × 350 mm with a spatial resolution of 2.6 × 2.6 mm. A half scan factor of 0.625 was used to achieve a scan duration of 284 ms, corresponding to a frame rate of 3.5 frames per second (fps). Representative bSSFP pSAT images are shown in Fig. 1, with the iSuite platform providing three simultaneous views of the balloon in the left ventricle.

### T1-overlay

**Fig. 2:**
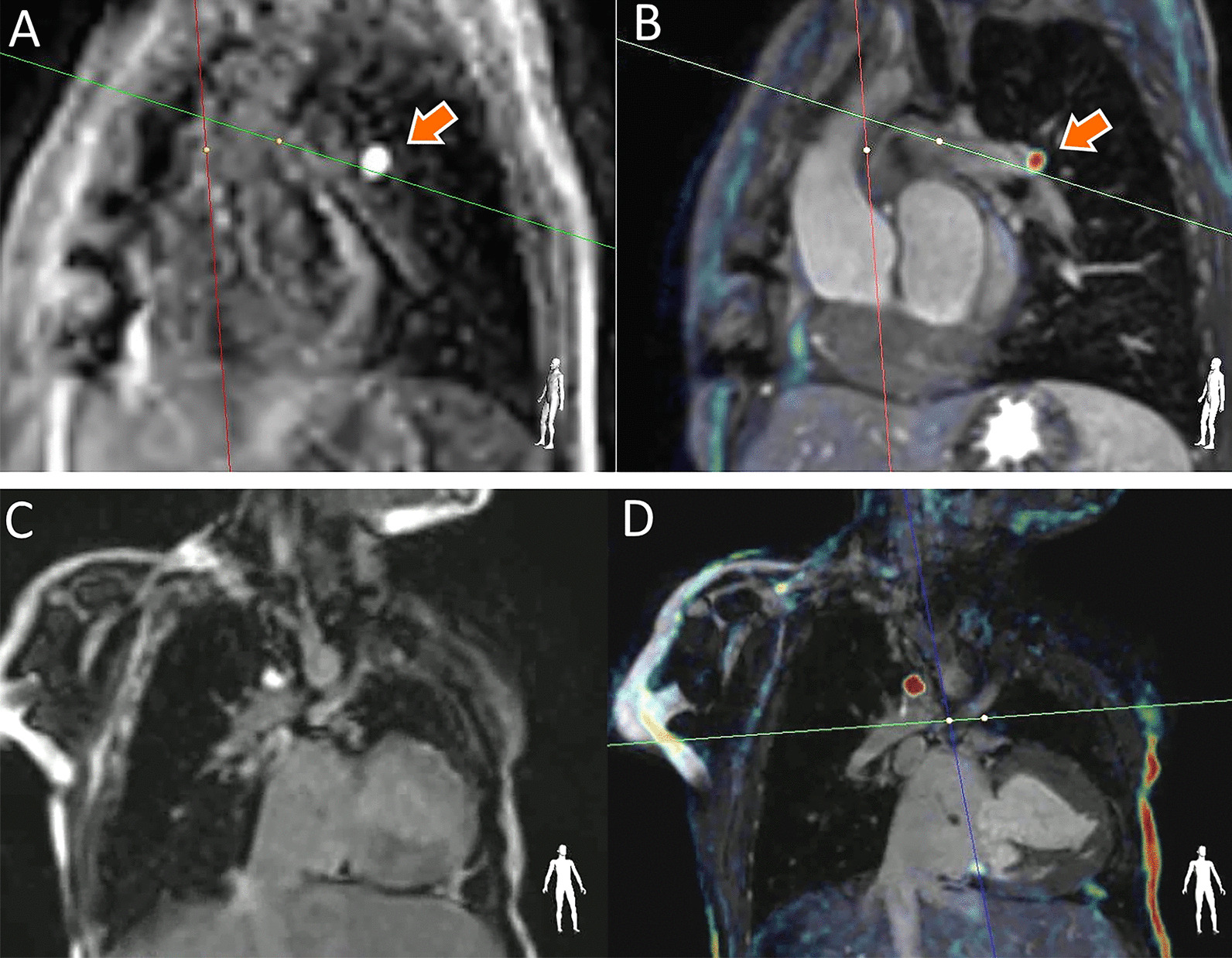
**A**, **B** A gadolinium-filled catheter in the left pulmonary artery of another patient. **A** The proposed real-time T1-weighted spoiled gradient echo image containing bright, isolated gadolinium filled balloon signal (orange arrow) with suppressed anatomical detail. **B** The real-time image from (**A**) with a manually-selected threshold applied to overlay the brightest (isolated catheter) signal on the high-resolution 3D dataset acquired at the beginning of the session. This provides the interventionalist with both high-resolution anatomical detail and clear visualization of the gadolinium- filled balloon wedge catheter tip. **C**, **D** The catheter in the Glenn anastomosis of a different patient using the bSSFP pSAT acquisition (**C**), and the proposed T1-overlay technique (**D**) demonstrating improved visualization of the catheter tip and anatomy. This improved catheter tracking can be further appreciated in Additional file 1, which shows the real-time T1-weighted images of the catheter in another patient during a right heart catheterization, and the resulting iSuite overlay that is projected into the scan room to guide the interventionalist

To simultaneously improve the visualization of the catheter tip and cardiac anatomy, heavily T1-weighted images were acquired using a spoiled gradient echo sequence with a pre-saturation pulse to provide distinct real-time signal from the gadolinium-filled balloon (Fig. 2A). The bright balloon signal was isolated using a manually adjusted overlay threshold after balloon insertion, where a colormap was applied to the highest signal intensities, and low signal intensities were made transparent. This isolated balloon signal was overlaid in real-time within iSuite on the high-resolution, static anatomical images reformatted from the previously acquired 3D whole-heart roadmap (Fig. 2B). The slice location and orientation of these real-time T1-weighted acquisition and the underlying bSSFP anatomical image are synchronized throughout the procedure to provide simultaneous visualization of the balloon and its location within the vasculature. The combined real-time balloon signal and static anatomy images were then projected into the scan room for guidance.

Because these heavily T1-weighted images suppress background tissue signal, and only provide real-time updates of the balloon’s location, a thicker imaging slab can be used to minimize the loss of visualization during through-plane movements of the balloon without sacrificing the excellent visualization of the underlying anatomy. A 20 mm slice thickness was used for this approach, in contrast to the 8 mm slice thickness of the bSSFP with pSAT acquisition. The real-time T1-overlay images were acquired using a spoiled gradient echo with a non-selective saturation pre-pulse, FOV = 350 × 350 mm, TE/TR = 1.3/2.6 ms, 45º flip angle, resolution = 2.6 × 2.6 mm, half-scan factor = 0.625, and acquisition duration of 318 ms corresponding to a frame rate of 3 fps.

### Real-time imaging sequence comparison

The performance of each sequence was evaluated in the first 30 patients by qualitative assessment of image quality, as well as the duration of time that the catheter was visible throughout the procedure by reviewing recordings of each case [8]. Image quality was scored based on the visualization of cardiac structures, contrast between the balloon and blood pool [11], and overall suitability of the technique for iCMR guidance (Table 1). Rating of image quality was independently performed by three cardiologists (T.H, G.G, S.R) with more than 10 years of experience with CMR and more than 5 years of experience with CMR-guided cardiac catheterization. Scores that differed by 2 points or more were reviewed and scored by consensus. To evaluate catheter visualization time, two authors (J.S.G, M.A.H.) reviewed each recording and noted the duration of time that the catheter was in view as a fraction of the total procedure duration.

**Table 1:** Image quality scoring system

Score	Visualization of cardiac structures	Blood pool and balloon contrast	Suitability for iCMR guidance
1—Poor	Unable to differentiate cardiovascular structures	Impossible to differentiate balloon and blood pool	Insufficient visualization of the balloon or cardiovascular structures to carry out the procedure
2—Low	Cardiovascular structures can be slightly intuited	Differentiation of blood and balloon difficult to perform	Challenging to navigate catheter due to insufficient image quality
3—Intermediate	Cardiovascular structures can be delineated	Adequate signal disparity	Sufficient visualization
4—Good	Good definition of cardiovascular structures	Differentiation of blood and balloon easily performed	Catheter easily navigated throughout procedure
5—Excellent	Optimal cardiovascular structure delineation	Excellent contrast between blood and balloon	High confidence in catheters location

*iCMR* interventional cardiovascular magnetic resonance

The inclusion of the 30 remaining T1-overlay cases would increase statistical power of these balloon visualization and image quality analyses. However, interim analysis of the first 30 cases showed such improved catheter tracking that further direct comparison of the two sequences was stopped in the interest of providing consistent visualization of the catheter tip. Therefore, in the second group of 30 patients, T1-overlay was preferentially used for guidance and the bSSFP sequence was only temporarily used when needed to position the guidewire. The number of such cases where bSSFP was required as backup was recorded in the second set of patients.

### Selective angiography

Selective angiography is an essential technique for the real-time evaluation of blood flow dynamics during conventional x-ray fluoroscopy cardiac catheterization, and is routinely used for the detection of coronary artery obstructions, aorto-pulmonary collaterals, and pulmonary AVMs. Patients with single-ventricle physiology tend to develop micro-AVMs, which are not well visualized by anatomical imaging. It is therefore common practice to indirectly identify AVMs by the pulmonary transit time, or the time it takes for a direct contrast injection in the pulmonary artery to arrive in the pulmonary vein (typically around 3–5 heartbeats), which is reduced in the presence of pulmonary AVMs. Because selective angiography is relied on to provide this diagnostic information, it is important that a suitable CMR alternative be developed and validated so that similar clinical questions can be answered during iCMR-guided cardiac catheterization.

CMR selective angiography has been previously demonstrated using catheter-directed gadolinium contrast injection in a limited number of subjects [16, 17], but has not been widely adopted for clinical applications. Because the T1-overlay approach provides excellent visualization of the gadolinium contrast agent within the vasculature, it is well-suited to provide real-time imaging of blood flow dynamics, analogous to fluoroscopic selective angiography. This technique gives anatomical context to the injected contrast agent to aid in interpretation during the procedure, and enables a thick slab acquisition for straightforward planning of the imaging plane to capture the contrast at both its injection site and its arrival in the pulmonary veins.

Selective angiography was performed in 23 of the 60 patients who underwent iCMR-guided catheterization to evaluate pulmonary transit times. Gadolinium contrast was diluted to 1/100 gadolinium/saline, and was administered as a 10–20 mL bolus in either the superior vena cava, branch pulmonary arteries, or Fontan conduit, so that contrast would flow directly into the pulmonary circulation. Sixteen of these patients were determined to require further intervention, such as Fontan fenestration device closure, balloon angioplasty, or coiling of collateral vessels, and were immediately transferred to the x-ray fluoroscopy lab after the iCMR procedure. Fluoroscopic selective angiography was also performed in these patients for the assessment of pulmonary transit time, providing a direct comparison of iCMR transit time measurements with the clinical standard.

Imaging of selectively-injected gadolinium contrast was performed using the same sequence parameters as the T1-overlay approach, described above, with the slice thickness increased to 60 mm to maintain visualization of contrast during complex through-plane motion. Pulmonary transit time was defined as the time when contrast was first be seen at the injection site until it arrived in the left atrium. CMR image timestamps allowed this period to be converted to the exact number of heartbeats using the patient’s heart rate. For x-ray fluoroscopy, transit time was recorded as a range, e.g. 4–5 beats, as timestamps were not available. For comparison to iCMR, this transit time was rounded to the midpoint of this range, e.g. 4.5 beats.

### Statistical analysis

Improvement in the catheter visualization time provided by T1-overlay compared to the bSSFP real-time sequence was evaluated by paired t-test, with p < 0.05 considered significant. The three image quality metrics (Table 1) for each sequence were evaluated by Wilcoxon signed rank test. Agreement between the iCMR and fluoroscopy estimates of pulmonary transit time were evaluated by Pearson correlation and Bland–Altman analysis.

## Results

Measurement of the predefined pressures and saturations using CMR for guidance was successful in all 60 patients. Figure 1 shows the iSuite platform with three views of the balloon wedge catheter in the ventricle using the pSAT acquisition. While this sequence provides the benefit of real-time imaging of the anatomy, the contrast between the blood pool and myocardium is limited, and the thin imaging slab required for adequate visualization of the vasculature often results in loss of balloon visualization as it is advanced through the vasculature.

### T1-overlay

**Fig. 3:**
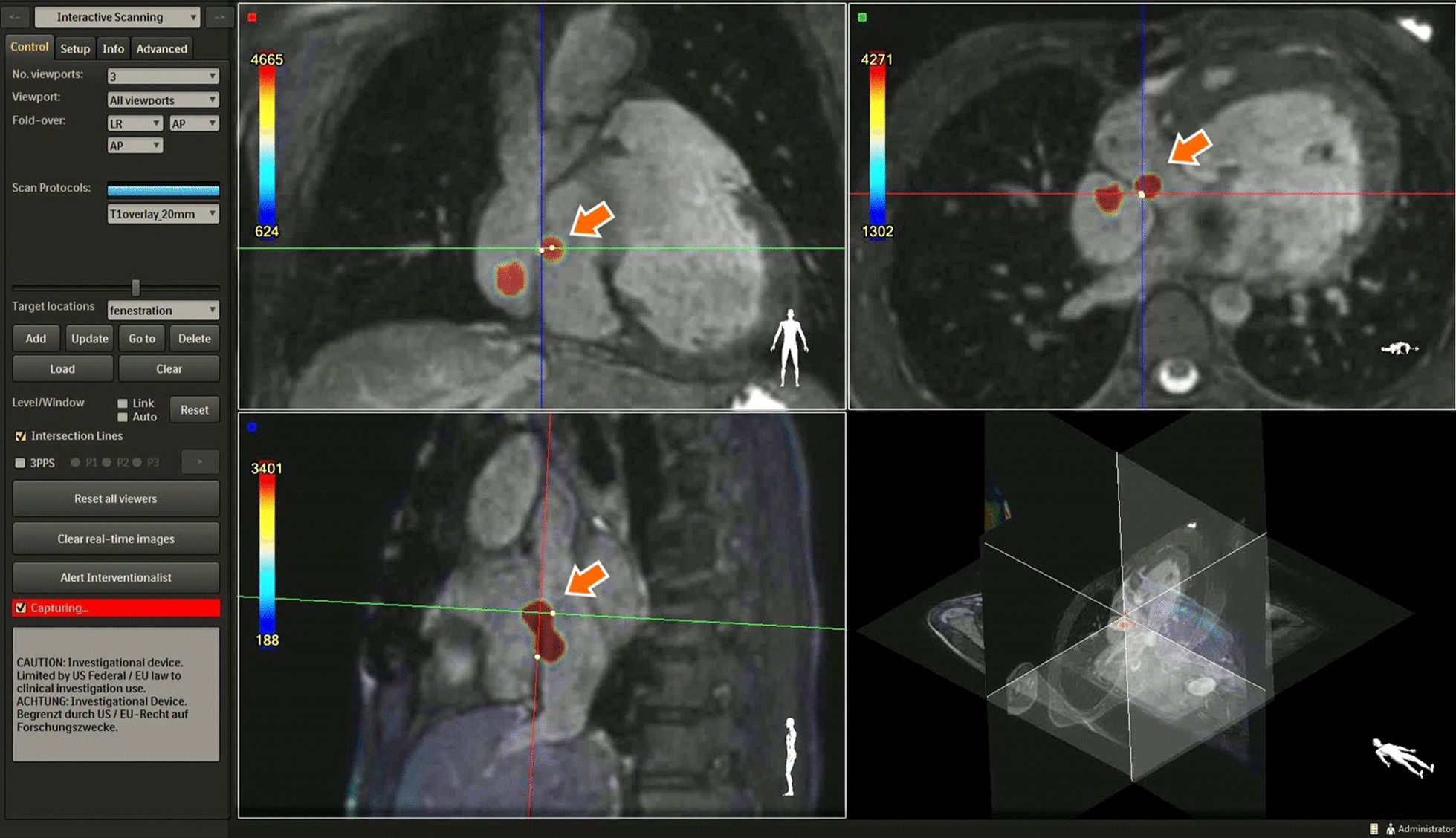
iSuite platform with the proposed T1-overlay approach during a Fontan fenestration test occlusion (FFTO). Two distinct gadolinium filled catheter tips shown in three orientations. One gadolinium filled balloon wedge catheter is shown closing the fenestration (orange arrow) while the other (shown in the Fontan conduit) is free to measure pressures and blood oxygen saturation during FFTO

Figure 2 shows the source T1-weighted images acquired for real-time navigation (A), and the combined overlay images shown to the interventionalist throughout the procedure (B). Figure 2C, D shows the catheter in the Glenn anastomosis of another patient using the pSAT bSSFP acquisition (C), and the T1-overlay technique (D) demonstrating improved visualization of the catheter tip and anatomy. Figure 3 shows the T1-overlay approach being used for a Fontan fenestration test occlusion in another patient [18].

**Fig. 4:**
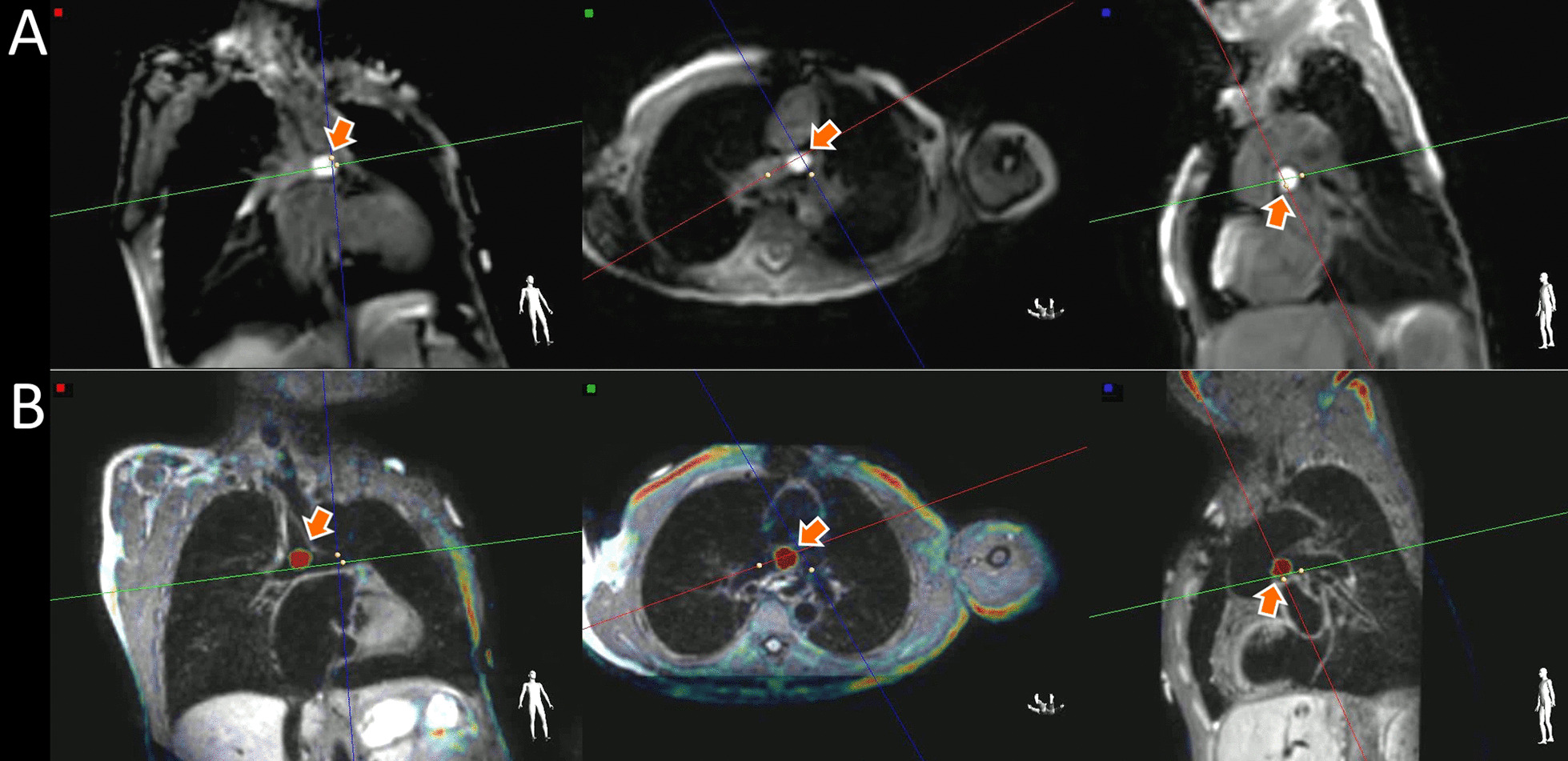
Images of the catheter in a patient with Glenn physiology, acquired using the pSAT approach (**A**) and T1-overlay on a 3D black-blood dataset (**B**) in three sequentially-acquired imaging planes. The 3D black-blood acquisition provides excellent visualization of the vessel walls, enabling the catheter to be clearly seen within the lumen of the vessel with T1-overlay. In some cases, using a 3D turbo spin echo (TSE) black-blood dataset as a roadmap may be beneficial for visualization of the lumen of the vasculature, or for improved imaging of the anatomy near metal implants. A short video of this case can be seen in Additional file 2, where the catheter is clearly seen as it is advanced from the left pulmonary artery (LPA) to the right pulmonary artery

**Fig. 5:**
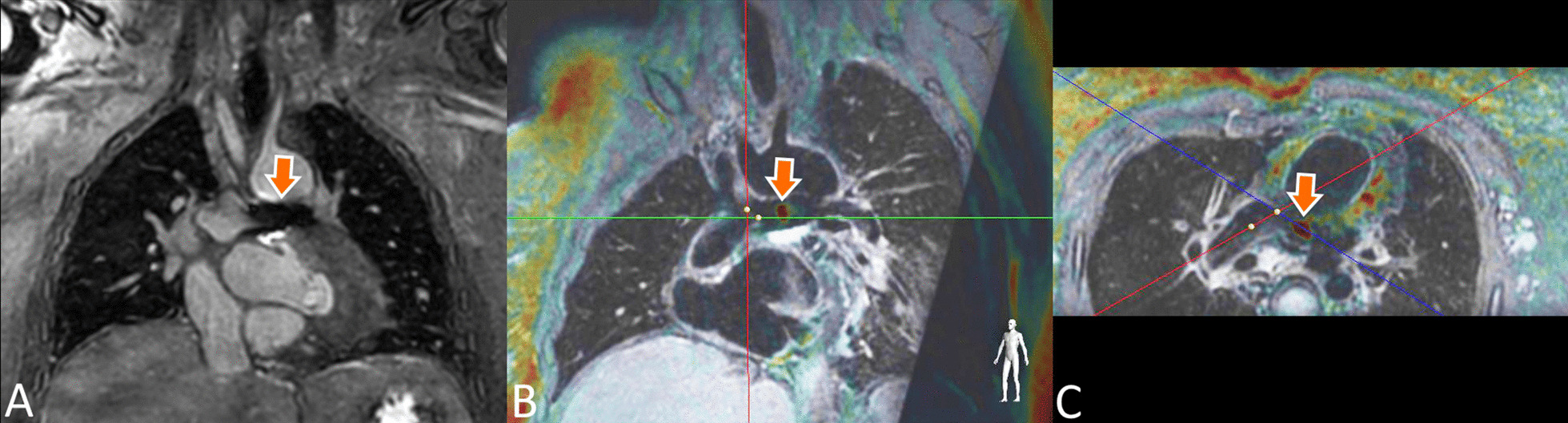
T1-overlay technique in a patient with an LPA stent. **A** The 3D whole-heart bSSFP with stent artifact (arrow) that would obstruct the view of the balloon using the bSSFP pSAT approach. **B**, **C** Two views of LPA stent using the B0-insensitive spoiled gradient echo, demonstrating that the balloon can still be tracked in regions of increased off-resonance

A 3D TSE black-blood dataset used for T1-overlay guidance is shown in Fig. 4B, where the catheter tip can be clearly seen in the lumen of the Glenn anastomosis. The pSAT approach (A) shows the balloon in the same location with a less detailed view of the anatomy. Because the T1-weighted spoiled gradient echo is less sensitive to field inhomogeneity than the bSSFP, it also results in improved visualization near stents and other metal implants. Figure 5 shows a patient with an left pulmonary artery stent with the bSSFP acquisition showing a significant signal void (A), while the gadolinium filled balloon can be seen passing through the stent with the T1-overlay approach.

### Real-time imaging sequence comparison

Across the first 30 cases, the balloon was in view for 55 ± 24% of the procedure with the pSAT sequence (mean ± standard deviation), and 89 ± 7% of the time with T1-overlay (Fig. 7). This approach significantly improves catheter visualization time (p < 0.001) and has made the balloon much easier to track throughout the procedure. All three measures of image quality were also significantly improved (*p* < 0.05) with T1-overlay approach (Fig. 7B).

### Guidewire visualization

In the second group of 30 patients in which T1-overlay was primarily used for guidance, 17 patients required the bSSFP acquisition to temporarily be used to position a guidewire for assistance with catheter navigation. Switching between these imaging sequences takes a few seconds within iSuite, so that the ideal sequence for a specific portion procedure can be easily selected.

### Selective angiography

**Fig. 6:**
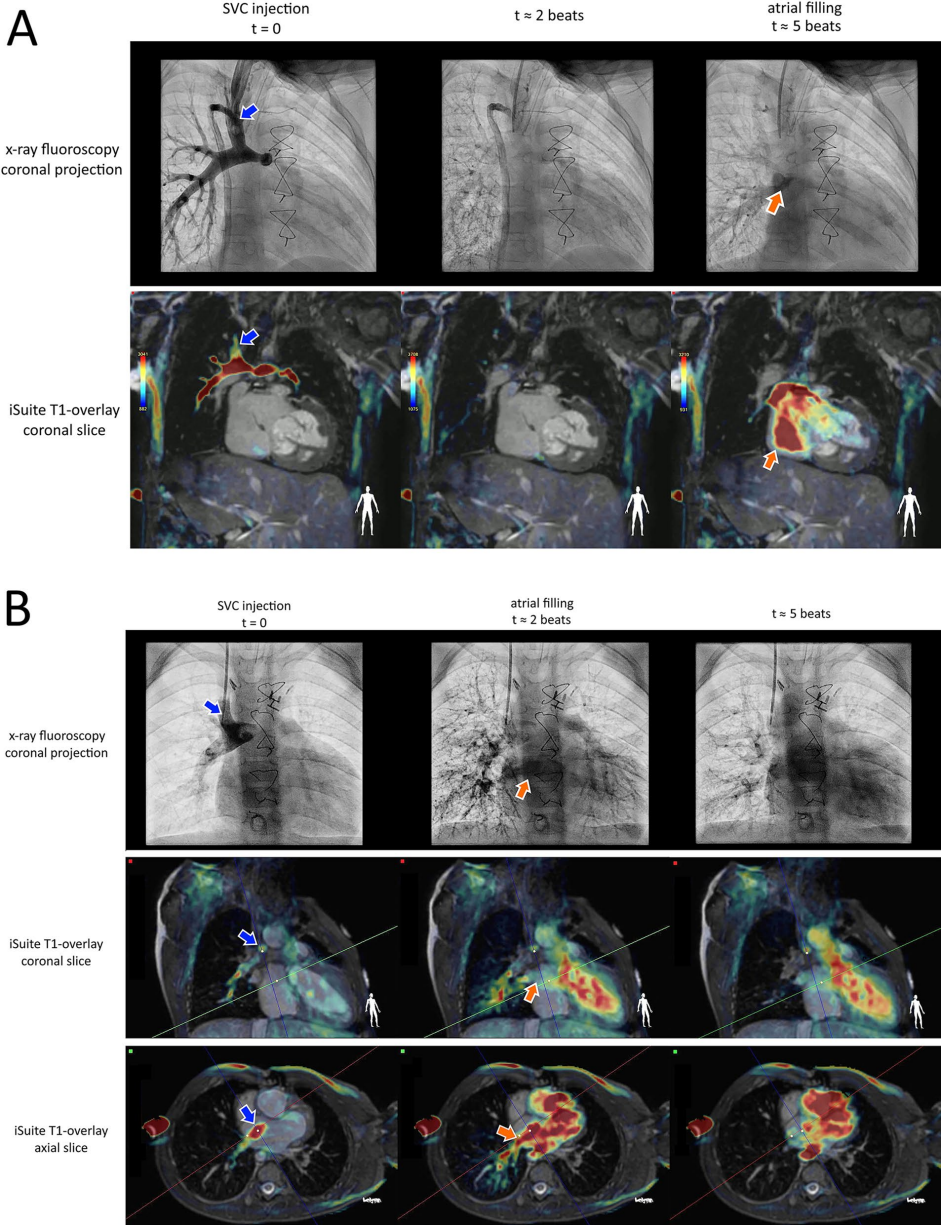
**A** Selective injection angiogram in a patient with a right sided Glenn with a normal pulmonary transit time. From left to right, the panels show the flow of the injected contrast agent from the superior vena cava (blue arrows), directly to the lungs, and back into the heart over several heartbeats with x-ray fluoroscopy and CMR in coronal planes. A manually-selected threshold was applied to the T1-weighted CMR to overlay the bright gadoliniumsignal on the high-resolution 3D dataset acquired at the beginning of the session, providing real-time visualization of the contrast flow patterns. In this patient (**A**), atrial filling (orange arrow) was not observed until the 5th heartbeat (third column) with both modalities, indicating normal transit time through the lungs. **B** Selective injection angiogram in another patient with a right sided Glenn (blue arrow) with T1-overlay images acquired in coronal and axial planes simultaneously. The second time point (middle column) shows rapid atrial filling (less than 2 heartbeats after the injection) with both modalities, suggesting the presence of pulmonary arteriovenous malformations (AVMs) in the right lung, which were confirmed by fluoroscopy in this patient. Videos of these selective angiograms can also be seen in Additional file 3

**Fig. 7:**
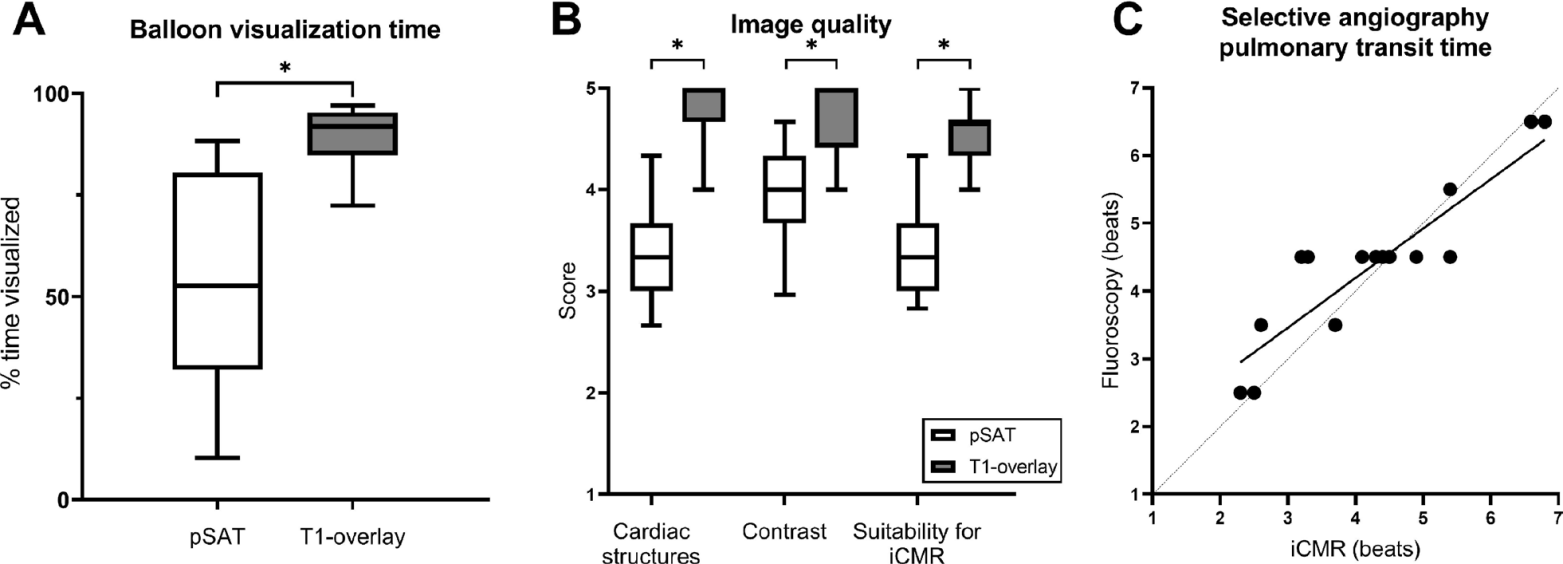
Results of the balloon visualization analysis (**A**), image quality rating (**B**), and agreement between pulmonary transit time measurements (**C**). T1-overlay significantly improved the amount of time that the balloon was kept in view throughout the procedure in the first 30 patients due to the high contrast images and thicker imaging slab (**A**), p < 0.001. T1-overlay images were also rated significantly higher in these patients in all three qualitative categories: visualization of cardiac structures (p < 0.05), blood/balloon contrast (p < 0.05), suitability for iCMR guidance (*p* < 0.05). The boxes (**A**, **B**) extend from the 25th to 75th percentiles, with a line at the median, and the whiskers to the minimum and maximum values. The measurements of pulmonary transit time (**C**) also agreed very well between fluoroscopy nd iCMR selective injections (r = 0.89)

Figure 6 shows the evaluation of pulmonary transit time using selective angiography in two pre-Fontan patients, with contrast injected in the Glenn anastomosis with x-ray fluoroscopy and iCMR T1-overlay in a patient with normal pulmonary circulation (A) and with pulmonary AVMs (B). The pulmonary transit time estimates in the 16 patients who received both iCMR and fluoroscopy selective injections agreed very well, indicating that iCMR selective angiography may be useful as a screening tool for pulmonary AVMs in the future. Bland–Altman analysis showed a bias of − 0.1 beats, with 95% limits of agreement of − 1.3 to 1.1 beats in these patients. Figure 7C shows the correlation between the fluoroscopy and iCMR estimates of pulmonary transit time (r = 0.89).

## Discussion

The T1-overlay technique developed in this study increases confidence in the location of the catheter and facilitates catheter guidance through complex anatomies. By acquiring a high-contrast image of the catheter tip and overlaying it on a high-resolution 3D whole-heart dataset, we significantly improved the ability of the technologist to maintain passive visualization of the catheter tip throughout the procedure compared to a standard bSSFP acquisition. The distinct balloon signal provided by the T1-weighted acquisition allows the use of a thicker imaging slice, which enables the operator to easily keep the balloon in plane without the need for active-tracking hardware development. Because the 3D bSSFP roadmap sequence is designed to provide excellent visualization of the vasculature with minimized motion artifacts, it is ideal for providing continuous high-resolution visualization of the anatomy. The ability to maintain catheter visualization, combined with the excellent tissue definition provided by the 3D roadmap dataset, may enable more complex procedures to be performed using this technique. The T1-overlay was also greatly favored by the interventionalist over the pSAT approach, as there was rarely any question about the location of the catheter tip, and was scored more favorably in all three qualitative metrics.

While this guidance technique is possible using a commercially available imaging sequence, it relies on the overlay feature of iSuite, which is an investigational device, and not yet approved for clinical use. Similar overlay approaches have also been developed to display projection images of catheterization devices on the real-time acquired images, analogous to x-ray fluoroscopy [19]. While our approach with selective excitations may be beneficial for localizing devices in 3 dimensions, overlaying on a static 3D dataset comes with the disadvantage of potentially outdated tissue context.

Although the proposed heavily T1 weighted sequence suppresses nearly all background tissue, some residual fat signal remains bright and is overlaid in the final image. This is particularly apparent in Fig. 5B, C. This bright subcutaneous fat has not presented any balloon visualization challenges in this study due to its location, although epicardial or pericardial fat could interfere with intracardiac balloon tracking using T1-overlay. In such cases, a fat suppression pre-pulse could be added to minimize this signal. Optimization of the real-time T1 weighted sequence is also warranted, as further reductions in specific absorption rate (SAR) (e.g. minimizing the saturation and acquisition flip angles) may be possible while still providing effective isolation of the gadolinium balloon. The sparsity of these real-time T1 weighted balloon images may also make them a good candidate for heavy acceleration through undersampling and compressed sensing reconstruction to further increase the frame rate.

The ability to visualize the location of the gadolinium contrast within the vasculature enabled the real-time visualization of selectively-injected contrast, allowing for more thorough evaluation of blood flow dynamics. This allowed for the measurement of pulmonary transit time using iCMR selective-angiography, which agreed well with similar measurements made by fluoroscopy. In addition to the detection of pulmonary AVMs (Fig. 6), we have identified the detection of Fontan fenestration flow as another potential application for this technique in our patients. Because the T1-overlay approach allows for the acquisition of thick real-time imaging slabs, the injected contrast agent can be visualized as it flows from the injection site to the pulmonary veins in a single slice. Although these 2D imaging slabs cannot resolve through-plane motion of the contrast, they allow a much higher frame rate than can be achieved with a 3D acquisition. For example, the temporal resolution of a 3D time-resolved angiogram is generally on the order of 6 s, whereas multiple 2D frames can be acquired per heartbeat for the evaluation of pulmonary transit time. Additionally, the iSuite system enables multiple views to be interleaved (B) for a more thorough representation of 3D flow dynamics if needed. Further translation of other x-ray fluoroscopy techniques into the iCMR suite, like selective angiography, could help accelerate the growth of iCMR-guided cardiac catheterization and transcatheter interventions.

### Limitations

A limitation of this technique is potential misregistration of the real-time balloon signal relative to the underlying static anatomical roadmap. In some cases, a catheter guidance technique that employs semi-regular anatomical imaging will be required to minimize safety concerns. For example, patients who are not under anesthesia may not remain perfectly still, and would therefore not be aligned with the static anatomical roadmap used to navigate the catheter. Cardiac interventions will also require real-time anatomical imaging to visualize the effects on the tissue. In this study, the 3D roadmap and catheterization procedure were performed sequentially under the same anesthetic conditions. Therefore, patient motion was minimized and significant misregistration of the catheter was not encountered in our experience.

Because guidewires and other interventional devices are generally visualized through bSSFP off-resonance artifacts [7], they are not visible with the current T1-overlay implementation. This reduced sensitivity of the spoiled gradient echo acquisition to off-resonance does, however, offer the benefit of improved tracking of the balloon near stents and other implanted devices (Fig. 5). In cases where guidewire visualization is required, the bSSFP sequence is currently needed. Techniques to generate positive contrast around paramagnetic devices have been proposed to visualize the guidewire with bSSFP [20], though this may be challenging to combine with T1-overlay. Nevertheless, the development of an approach to visualize both interventional devices and isolated balloon signal would be a significant benefit, as the spoiled gradient echo acquisition offers reduced SAR compared to the bSSFP pSAT approach with superior tracking of the balloon. This could significantly reduce device heating, and would enable additional devices to be used for iCMR-guided interventions.

An ideal comparison of the procedure duration using the two imaging approaches would be timed serial catheterization in each patient, with the order of sequences being randomized and fresh catheters used for each to minimize the effects of heating. However, for appropriate clinical care of these young patients, we made every attempt to minimize anesthesia time. Hence, we chose the current methodology wherein the imaging sequence was randomized to different parts of the procedure. Furthermore, the percent time that the catheter is visualized has previously been used as a measure of improved catheter guidance, as it is widely recognized as a main limitation for the safety and speed of these procedures [8].

## Conclusion

We have demonstrated improved visualization of the catheter tip using T1-overlay, resulting in a significant increase in the time that the operator was able to maintain visualization of the catheter. We also demonstrated the initial feasibility and clinical utility of iCMR selective angiography using this approach to expand this fluoroscopic technique into the CMR environment.

## Supplementary Information

**Additional file 1.** Video of the real-time source T1-weighted images (left) and the corresponding iSuite display with real-time images overlaid on the 3D whole-heart dataset (right) during a right-heart catheterization. When the axial imaging plane is enabled, the acquisitions of the two geometries are interleaved, shown by the rapidly switching coronal and axial source images. These images are overlaid on the corresponding anatomical images within iSuite, providing the interventionalist with multiple real-time views of the catheter

**Additional file 2.** Video of the T1-overlay approach used with a 3D black-blood dataset in a patient with Glenn physiology. The balloon can be clearly followed within the lumen of the vasculature as it is pulled into the superior vena cava from the left pulmonary artery, then advanced into the right pulmonary artery.

**Additional file 3.** Synchronized fluoroscopy and iCMR selective angiograms in the same Glenn patient. Contrast was injected into the superior vena cava and flowed across the pulmonary circulation after 4–5 heartbeats.

## Data Availability

The datasets used and/or analyzed during the current study are available from the corresponding author on reasonable request.

## Abbreviations

AVMs: Arteriovenous malformations
bSSFP: Balanced steady state free precession
CMR: Cardiovascular magnetic resonance
FFTO: Fontan fenestration test occlusion
iCMR: Invasive cardiovascular magnetic resonance
LPA: Left pulmonary artery
pSAT: Partial saturation
SAR: Specific absorption rate
SNR: Signal-to-noise ratio
TSE: Turbo spin echo
